# Structural analysis of DNA binding by C.Csp231I, a member of a novel class of R-M controller proteins regulating gene expression

**DOI:** 10.1107/S139900471402690X

**Published:** 2015-01-23

**Authors:** M. B. Shevtsov, S. D. Streeter, S.-J. Thresh, A. Swiderska, J. E. McGeehan, G. G. Kneale

**Affiliations:** a Biophysics Laboratories, Institute of Biomedical and Biomolecular Sciences, School of Biological Sciences, University of Portsmouth, Portsmouth PO1 2DY, England

**Keywords:** DNA–protein interactions, helix–turn–helix, restriction–modification, controller protein, gene regulation, EMSA

## Abstract

In a wide variety of bacterial restriction–modification systems, a regulatory ‘controller’ protein (or C-protein) is required for effective transcription of its own gene and for transcription of the endonuclease gene found on the same operon. We have recently turned our attention to a new class of controller proteins (exemplified by C.Csp231I) that have quite novel features, including a much larger DNA-binding site with an 18 bp (∼60 Å) spacer between the two palindromic DNA-binding sequences and a very different recognition sequence from the canonical GACT/AGTC. Using X-ray crystallography, the structure of the protein in complex with its 21 bp DNA-recognition sequence was solved to 1.8 Å resolution, and the molecular basis of sequence recognition in this class of proteins was elucidated. An unusual aspect of the promoter sequence is the extended spacer between the dimer binding sites, suggesting a novel interaction between the two C-protein dimers when bound to both recognition sites correctly spaced on the DNA. A U-bend model is proposed for this tetrameric complex, based on the results of gel-mobility assays, hydrodynamic analysis and the observation of key contacts at the interface between dimers in the crystal.

## Abstract 2

The structure of the new class of controller proteins (exemplified by C.Csp231I) in complex with its 21 bp DNA-recognition sequence is presented, and the molecular basis of sequence recognition in this class of proteins is discussed. An unusual extended spacer between the dimer binding sites suggests a novel interaction between the two C-protein dimers.

## 1. Introduction   

Restriction–modification (R-M) systems protect bacteria from invasion by foreign DNA. They are involved in the horizontal transfer of genes in bacterial populations, and may play a role in the spread of antibiotic-resistant genes (Loenen *et al.*, 2014 ▶; Lindsay, 2010 ▶; Waldron & Lindsay, 2006 ▶; Kobayashi, 2001 ▶; Akiba *et al.*, 1960 ▶). R-M systems employ a variety of mechanisms to ensure the correct temporal regulation of the methyltransferase and restriction-endonuclease genes. This is frequently achieved by controller proteins (C-proteins) that regulate the transcription of the R-M genes (Tao *et al.*, 1991 ▶; Ives *et al.*, 1992 ▶; Rimšeliené *et al.*, 1995 ▶; Vijesurier *et al.*, 2000 ▶; Cesnaviciene *et al.*, 2003 ▶; Knowle *et al.*, 2005 ▶). Restriction-endonuclease activity must be delayed until the host DNA has been protected from cleavage by the cognate DNA methyltransferase, which methylates specific sites in the bacterial genome and prevents cleavage by the endonuclease. If this temporal control mechanism is impaired, this leads to degradation of the host genome and results in cell death (Mruk & Blumenthal, 2008 ▶; Enikeeva *et al.*, 2010 ▶). Our goal is to understand the structure and mechanism of such control systems.

A bioinformatics study has identified many hundreds of potential C-protein genes in the DNA sequence database (Sorokin *et al.*, 2009 ▶). However, only a fraction of these genes have been shown to encode functional proteins, and even fewer of these have been the subject of structural or biophysical analysis. C-proteins have been divided into several classes based on motifs in their predicted DNA-recognition sites and/or the amino-acid sequences of the proteins (Sorokin *et al.*, 2009 ▶; Mruk *et al.*, 2007 ▶). X-ray crystallographic and functional information now exists for the controller proteins of AhdI (McGeehan *et al.*, 2004 ▶, 2005 ▶, 2006 ▶; Bogdanova *et al.*, 2008 ▶), BclI (Sawaya *et al.*, 2005 ▶) and Esp1396I (Ball *et al.*, 2009 ▶; McGeehan *et al.*, 2008 ▶, 2012 ▶; Bogdanova *et al.*, 2009 ▶; Martin *et al.*, 2013 ▶, 2014 ▶). Other systems such as PvuII, although extensively studied *in vitro* and *in vivo* (Rimšeliené *et al.*, 1995 ▶; Vijesurier *et al.*, 2000 ▶; Mruk *et al.*, 2007 ▶), have not been studied at a detailed structural level. Together, these studies have revealed a highly cooperative, concentration-dependent genetic switch which ensures that expression of the endo­nuclease is delayed until the methyltransferase has been produced (Streeter *et al.*, 2004 ▶; Mruk *et al.*, 2007 ▶; McGeehan *et al.*, 2008 ▶; Bogdanova *et al.*, 2008 ▶, 2009 ▶).

Upstream of the C-gene, the majority of such R-M systems have two C-protein binding sites, usually quasi-palindromic, having the consensus sequence **GACT**TAT**AGTC** but with variations on this motif (Ives *et al.*, 1992 ▶; Ball *et al.*, 2012 ▶). The two dimer-binding sites on the DNA are typically separated by ∼4 bp, such that the two C-protein dimers overlap on opposite sides of the DNA helix and interact across the major groove (McGeehan *et al.*, 2008 ▶). To date, all structural studies have been confined to this class of C-protein.

**Figure 1:**
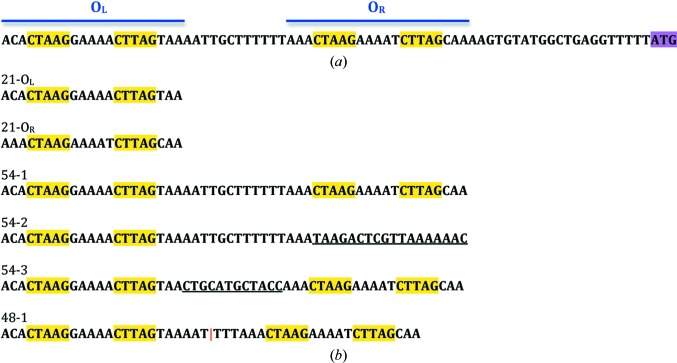
Upstream DNA sequences. (*a*) DNA sequence of the C-protein binding site upstream of the C-gene in Csp231I, showing the two operator sites, O_L_ and O_R_ (blue), and highlighting the inverted repeats (yellow) and the start codon of the C-gene (magenta). (*b*) DNA sequences used in experiments: variants (underlined) of the native operator sequence (54-1) include randomization of the O_R_ sequence (54-2) or of the central spacer (54-3). The sequence 48-1 corresponds to a deletion of the central six bases in the spacer; the deletion site is indicated by the red line. For simplicity, only one strand of the DNA is shown for all duplexes.

However, bioinformatic analysis revealed additional classes of C-proteins based on a variety of distinct DNA-recognition sites (Sorokin *et al.*, 2009 ▶), and 517 putative C-proteins have now been reported in ReBase (http://rebase.neb.com/cgi-bin/azlist?cp). The control regions of the R-M systems classified by Sorokin and coworkers as motif 8 [typified by EcoO1091I (Imasaki *et al.*, 2004 ▶; Kita *et al.*, 2002 ▶) and Csp231I (Streeter *et al.*, 2009 ▶; McGeehan *et al.*, 2011 ▶)] have very different sequence motifs to those previously studied. In C.Csp231I, this region consists of two sets of 5 bp palindromic sequences with a 5 bp spacer (Fig. 1 ▶ *a*). C.Csp231I has the recognition sequence **CTAAG**N_5_ **CTTAG**, in which the inverted repeat sequences are separated by A-rich pentanucleotides (GAAAA and AAAAT, respectively, for the distal and proximal operators O_L_ and O_R_). The distance separating the two 15 bp recognition sites (18 bp) is much longer than the ∼4 bp sequence found in the systems that have previously been studied, in which the binding sites partially overlap. For the related system EcoO109I the spacer between the palindromic operators is even longer (25 bp). Thus, in neither case can the protein dimers interact on the DNA in the manner of other C-protein systems, and an alternative mode of interaction is required for this class of R-M controller proteins.

The C.Csp231I controller protein (*M*_r_ = 11 360) is ∼30% larger than those for which structures have been investigated to date (*e.g.* C.AhdI and C.Esp1396I). Comparison of the 98-amino-acid sequence of C.Csp231I with C.AhdI shows only 29% identity over 62 core residues, with C.Csp231I having a 32-amino-acid extension at the C-terminus to form two additional helices (McGeehan *et al.*, 2011 ▶). In contrast, C.Csp231I and C.EcoO109I share almost 70% sequence identity over the first 80 amino-acid residues, consistent with their almost identical DNA-recognition sites (McGeehan *et al.*, 2011 ▶).

In order to further our understanding of this group of transcriptional regulators, we embarked on structural and functional analysis of this member of a new class of C-proteins bound to various DNA sites corresponding to regions of the operator region upstream of its own gene. Here, we present the X-ray crystal structures of these DNA–protein complexes, together with analysis of the DNA-binding properties of C.Csp231I by electrophoretic mobility-shift assays (EMSAs) and analytical ultracentrifugation, leading to an understanding of the molecular interactions responsible for DNA-sequence recognition and a novel model for the tetrameric protein–DNA complex at the promoter site.

## 2. Materials and methods   

### 2.1. Expression, purification and crystallization   

The cloning and purification of the C.Csp231I protein from *Citrobacter* sp. RFL231 have been described previously (Streeter *et al.*, 2009 ▶). For crystallization trials, the protein was dialysed against buffer consisting of 50 m*M* Tris–HCl pH 8.0, 0.1 *M* NaCl, 1 m*M* Na_2_EDTA. HPLC-purified DNA oligonucleotides were purchased from ATDBio. DNA duplexes were prepared in 10 m*M* Tris–HCl pH 8.0, 0.1 *M* NaCl, 1 m*M* EDTA. The duplexes were annealed by heating to 95°C for 5 min and then slowly cooled to room temperature over a period of 12–15 h. The annealed duplexes were purified using a Superdex 200 10/300 GL (25 ml) size-exclusion column and concentrated using Vivaspin concentrator columns.

The protein–DNA complexes were prepared by mixing protein and DNA in various ratios, followed by incubation at room temperature for 30 min. Crystallization conditions were screened with the aid of a Honeybee X8 nanodrop robot (Digilabs) by sitting-drop vapour diffusion using the PACT screening kit (Molecular Dimensions). The prepared protein–DNA complex was mixed in a 1:1 ratio with the reservoir solutions (0.1 µl + 0.1 µl) and incubated at 289 K for several days. Further crystal-growth optimizations were performed manually employing the hanging-drop vapour-diffusion method. Several different oligonucleotide constructs with varying lengths were used in crystallization trials.

### 2.2. X-ray data collection, phasing and structure refinement   

Prior to cryocooling in liquid nitrogen, the crystals were cryoprotected by transfer into a solution containing the same components as the well solution with an increase of 3–5% in the precipitant and the addition of 15–20%(*v*/*v*) glycerol. The crystals were cryocooled in liquid nitrogen following mounting on cryoloops. Diffraction data for two crystal forms of the O_L_ 21-mer DNA–protein complex were collected on beamline ID14-4 at ESRF, France equipped with an ADSC Q315r X-ray detector. The crystals were maintained at 100 K and data were collected at a wavelength of 0.9393 Å with an oscillation width of 1.0° for monoclinic data or 0.5° otherwise. Data for one crystal form of the O_R_ 21-mer DNA–protein complex were collected on beamline I02 at the Diamond Light Source, UK. The crystals were maintained at 100 K using an Oxford Instruments Cryojet XL and data were collected at a wavelength of 0.9795 Å with an oscillation width of 1.0° using an ADSC Q315 CCD detector.

All crystallographic data were processed with *iMosflm* (Leslie, 1992 ▶) and *SCALA* (Evans, 2006 ▶). Data-collection and processing statistics for all crystal forms are given in Table 1 ▶. The scaled data were phased by molecular replacement with *Phaser* (McCoy *et al.*, 2005 ▶) using a dimer of C.Csp231I as a search model (PDB entry 3lis; McGeehan *et al.*, 2011 ▶). From these initial phases, the DNA duplexes were fitted by iterative rounds of building and refinement in *Coot* (Emsley & Cowtan, 2004 ▶) and *REFMAC*5.5 with TLS restraints enabled (Murshudov *et al.*, 2011 ▶). The first crystal form of the O_L_ 21-mer DNA–protein complex was found to be twinned (twinning fraction of 0.3). In this case, the amplitude-based twinning refinement option implemented in *REFMAC* was used. During the refinement of the O_R_ complex, we located two iodide ions in the structure (see Supplementary Fig. S1). Similar binding sites for iodide ions have been observed in a number of structures (Abendroth *et al.*, 2011 ▶).

Stereochemical quality was analysed using *PROCHECK *(Laskowski *et al.*, 1993 ▶), and coordinate and structure-factor files have been deposited in the Protein Data Bank with accession codes 4jcx, 4jcy and 4jqd. Biological interfaces were analysed using *PISA* (Krissinel & Henrick, 2007 ▶). Structural parameters of the bound DNA duplexes were analysed using *CURVES* (Lavery *et al.*, 2009 ▶). All structural figures were produced using *PyMOL* (Schrödinger).

### 2.3. Electrophoretic mobility shift assays (EMSAs)   

EMSAs were performed using nondenaturing gel electrophoresis. Complementary DNA strands corresponding to the sequence upstream of the C.Csp231I gene were purchased (ATDBio) and were annealed to form a duplex. Different molar ratios of the protein and DNA duplexes were incubated in binding buffer (10 m*M* Tris–HCl pH 8.0, 0.1 *M* NaCl, 1 m*M* EDTA) at room temperature for 30 min. The samples were loaded onto a 1 h pre-run 6.5% native polyacrylamide gel and run at room temperature in 0.25× TBE at 100 V for 100 min. The gels were stained with ethidium bromide and were then scanned using a G-Box imaging system (SynGene).

Several different oligonucleotide constructs were used to test the DNA-binding properties of the protein, including the normal 54-mer sequence and modified 54 bp sequences in which the second DNA-binding site or the linker region was mutated or deleted (Fig. 1 ▶ *b*). The concentration of the DNA duplex was kept constant at 2 µ*M* while adding increasing amounts of protein to reach the required molar ratios.

### 2.4. Analytical ultracentrifugation   

For analytical ultracentrifugation, samples were dialyzed against a buffer consisting of 10 m*M* Tris–HCl pH 8.0, 100 m*M* NaCl, 1 m*M* EDTA using Slide-A-Lyzer MINI dialysis units (Thermo Scientific). The 56-mer DNA duplex (Fig. 1 ▶) was used to study the interaction with the C.Csp231I protein. Sedimentation-velocity experiments were performed in a Beckman XL-A analytical ultracentrifuge equipped with an An50-Ti rotor. Double-sector Epon cells with path lengths of 1.2 cm were used with quartz window assemblies. The volume of loaded sample was 400 µl and the corresponding sample buffer volume was 420 µl. Samples were equilibrated at 20°C for 30 min and then accelerated to 20 000 rev min^−1^. Radial scans were performed at 10 min intervals at 260 nm. The DNA concentration was 0.76 µ*M* and for the tetrameric complex the protein was at a 4:1 molar ratio (subunits per DNA duplex). The partial specific volume for C.Csp231I was calculated from the amino-acid composition using *SEDNTERP* (Laue *et al.*, 1992 ▶) at 0.7448 ml g^−1^, with a buffer density of 1.00283 g ml^−1^ and a viscosity of 0.010137 P. Analysis of the scans was performed using *SEDFIT* (Schuck, 2000 ▶) to produce a distribution plot [*c*(*S*)] of the sedimentation-coefficient profile.

## 3. Results   

### 3.1. Crystallization of protein–DNA complexes   

Several different oligonucleotide constructs were used in crystallization trials. The best diffracting crystals were obtained using 21 bp duplexes corresponding to the core sequences of the O_L_ and O_R_ operator DNA (Fig. 1 ▶). Two crystal forms were obtained for the O_L_ complex, depending on the crystallization conditions, with the best diffracting crystals obtained using the following conditions: (i) buffer 1 [0.2 *M* ammonium chloride, 0.1 *M* MES pH 6.0, 20%(*w*/*v*) PEG 6K; protein (subunit):DNA molar ratio 1:1; protein concentration 1.2 mg ml^−1^; PACT condition B8], which produced a hexagonal form (*P*6_1_), and (ii) buffer 2 [0.2 *M* sodium nitrate, 0.1 *M* bis-tris propane pH 7.5, 24%(*w*/*v*) PEG 3350; protein (sub­unit):DNA molar ratio 2:1, protein concentration 1.5 mg ml^−1^], which produced a monoclinic form (*C*2). For the O_R_ complex, the optimum crystallization conditions were 0.2 *M* sodium iodide, 0.1 *M* bis-tris propane pH 6.0, 15%(*w*/*v*) PEG 3350, 10 m*M* spermidine; protein (subunit):DNA molar ratio 2:1; protein concentration 4.6 mg ml^−1^, which produced a hexagonal form (*P*6_1_).

### 3.2. Comparison of the three crystal forms   

The C.Csp231I–21-mer DNA duplex complexes crystallized in three crystal forms. For the O_L_ complexes (space groups *P*6_1_ and *C*2), the resolutions obtained were 2.30 and 2.75 Å, respectively, while the O_R_ (*P*6_1_) crystal form diffracted to a resolution of 1.80 Å (see Table 1 ▶). Molecular-replacement methods were used to phase all crystal forms using PDB entry 3lis (McGeehan *et al.*, 2011 ▶) as the search model. The structures of the two O_L_ DNA–protein complexes that crystallized in space group *C*2 (two complexes per asymmetric unit) are very similar to the *P*6_1_ O_L_ structure. We note here that the intermolecular interactions observed between the two complexes in the asymmetric unit of the *C*2 crystal form may be of biological significance (this is further elaborated in §4.4).

A comparison between the O_L_ and O_R_ structures reveals minor conformational differences, and these are principally in the flexible C-terminal region (residues 86–95); the latter are unlikely to be significant given the flexibility of this region of the protein. The overall r.m.s.d. between the two complex structures is 0.4 Å (or 0.3 Å if the flexible C-terminal region is excluded); subsequent analysis of the interactions of C.Csp231I with DNA is therefore based only on the O_R_ complex, since this has the highest resolution (1.8 Å) and the lowest *R* factor (*R*_work_ and *R*_free_ of 13.5 and 14.8%, respectively).

### 3.3. Overall structure of the complex   

**Figure 2:**
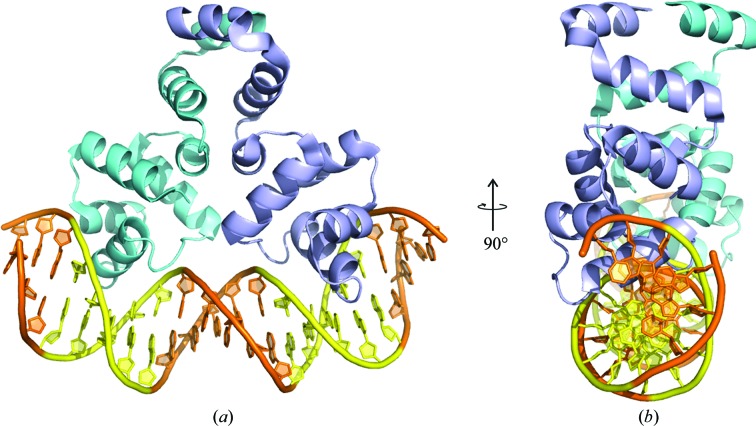
Overall structure of the C.Csp231I–DNA complex. (*a*) The protein dimer (cyan and violet subunits) bound to a DNA duplex (orange). The specific DNA-recognition sites (yellow) are located on both sides of the central pentanucleotide spacer. (*b*) Orthogonal view of the structure in (*a*).

The overall structure of the complex (Fig. 2 ▶) consists of a C-protein dimer bound to a DNA duplex. The structure of the free C.Csp231I protein dimer contains seven helices, as found in the free protein (McGeehan *et al.*, 2011 ▶), but with subtle conformational differences in the DNA-bound form of the protein. In the complex, each subunit interacts with the DNA by inserting recognition helix 3 (residues 28–40) of the classical helix–turn–helix motif into the major groove of the DNA either side of the central GAAAA motif. Superposition of monomer-to-monomer main-chain atoms reveals only minor differences when comparing subunits within the dimer. The maximum displacement between the main-chain atoms of separate monomers is confined to the C-terminal region (residues 86–95) of the protein (1.1 Å). This difference reflects conformational flexibility in this region of the protein, which has elevated values of crystallographic temperature (*B*) factors (see Supplementary Fig. S2). The observed flexibility of the C-terminal domain is similar in magnitude to that of the free protein structure (McGeehan *et al.*, 2011 ▶).

### 3.4. DNA-binding studies   

**Figure 3:**
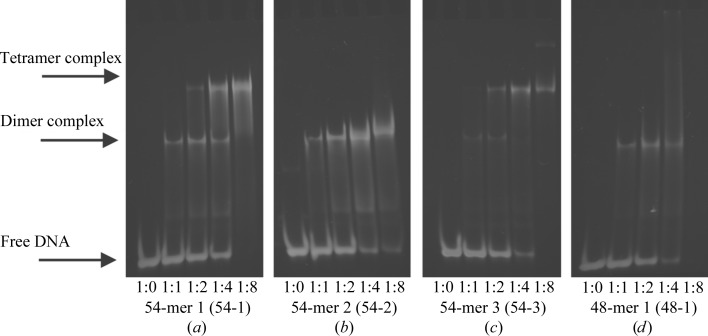
DNA-binding analysis. EMSAs showing the binding of C.Csp231I to various DNA sequences: (*a*) native 54 bp (54-1), (*b*) a 54-mer with a random second site (54-2), (*c*) a 54-mer with a random spacer (54-3) and (*d*) a 54 bp DNA fragment lacking the central 6 bp spacer (48-1). Precise sequences of the oligonucleotide duplexes are shown in Fig. 1 ▶. DNA duplexes were incubated at protein (subunit):DNA molar ratios of 0, 1, 2, 4 and 8 in each case. The DNA concentration was maintained at 2 µ*M* throughout.

EMSA analysis of C-protein binding to the left and right operators showed no differences in affinity between the two sites (data not shown). We thus investigated the interaction of C.Csp231I with longer DNA sequences corresponding to the 54 bp region encompassing the operator sites upstream of the C-gene (Fig. 1 ▶). EMSA experiments using the wild-type 54 bp fragment (Fig. 3 ▶ *a*) revealed a single complex at ratios of up to 2:1 (protein subunits per DNA duplex). At ratios of 4:1 and above a larger complex becomes apparent. These species are most likely to correspond to one and two bound dimers, respectively: one dimer bound at each palindromic recognition site of the DNA. We also looked at binding to an equivalent 54 bp oligonucleotide in which the sequence of the right-hand operator had been randomized (see Fig. 1 ▶). It is clear that mutation of this binding site blocks formation of the second species, suggesting that now only a single dimer binds to the wild-type O_L_ site (Fig. 3 ▶ *b*).

We then mutated the central spacer that is located between the dimer binding sites (see Fig. 1 ▶) to observe the effect of the DNA sequence of the spacer on protein binding. We found that random mutation of a 12 bp section of the central spacer had little effect on DNA binding (Fig. 3 ▶ *c*); however, a shorter (48 bp) oligonucleotide duplex in which the central 6 bp of the spacer had been removed allowed the binding of one protein dimer but prevented the binding of a second dimer (Fig. 3 ▶ *d*). Thus, the length of spacer is critical for binding two protein dimers simultaneously on the DNA, while the precise sequence of the spacer DNA appears to be unimportant. This suggests that there may be a structural role for the central spacer, rather than any sequence-specific interaction of the protein with the DNA bases in the spacer.

## 4. Discussion   

### 4.1. Conformation of bound DNA   

**Figure 4:**
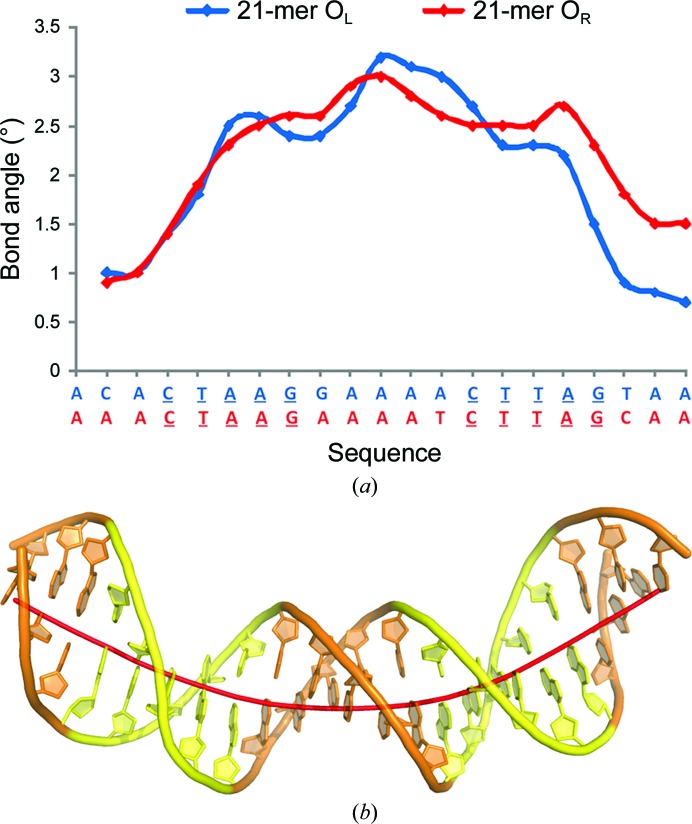
Structural distortions in bound DNA. (*a*) The local bend angle for 21-mer O_L_ (blue) and O_R_ (red) operators between adjacent base pairs (calculated as the angle formed between the normals of adjacent base pairs) is greatest at the central spacer sequence. Their sequences are shown below with the inverted repeats underlined. (*b*) Graphical representation of duplex bending. The overall DNA bend angles are 39 and 43° for the O_L_ and O_R_ duplexes, respectively

The DNA duplex in both complexes is significantly distorted from canonical B-form DNA (Fig. 4 ▶), with a bend of 39 and 43° for the complexes with O_L_ and O_R_, respectively, similar to the value (41°) observed in complexes of C.Esp231I with the O_L_ operator (McGeehan *et al.*, 2012 ▶). The bend angle induced in DNA when bound to the related C.EcoO109, as estimated from gel assays, was reported to be 54° (Kita *et al.*, 2002 ▶), although the results are not strictly comparable as they were obtained using different techniques.

The bend is stabilized by amino-acid contacts to the DNA backbone: principally electrostatic interactions with the phosphate groups on either side of the recognition site. It can be seen from the crystal structure of the DNA–protein complex that the charged and/or polar amino-acid side chains of Arg10, Gln17, Ser30, Arg34, Asn36, Tyr38, Lys40, Lys42 and His43 of each subunit interact electrostatically with the phosphodiester backbone at each half-site. These interactions are responsible in large part for contracting the minor groove of the DNA, which drives DNA bending of the DNA–protein complex.

### 4.2. Comparison of bound and free protein structures   

**Figure 5:**
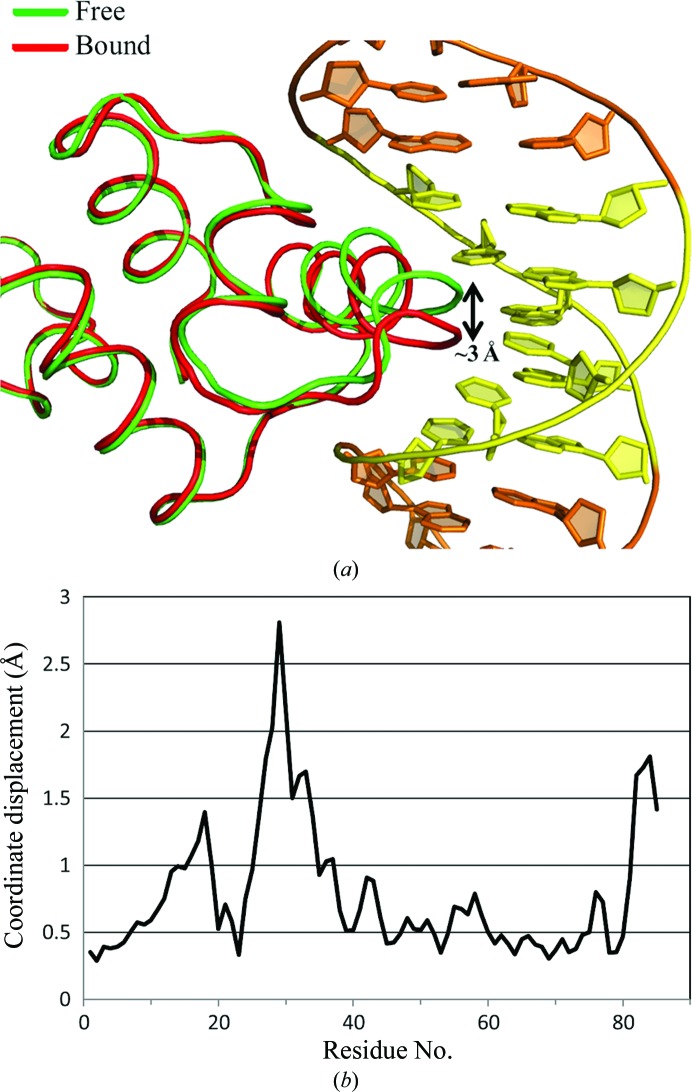
Comparison of free and DNA-bound protein subunit conformations. (*a*) The DNA-bound protein is shown in red for comparison with the structure of the free protein (PDB entry 3lis), shown in green. (*b*) R.m.s. deviation profiles showing quantitative differences between DNA-bound and free protein structures. The maximum observed displacement of the recognition-helix region (residues 28–40) upon DNA binding is 2.8 Å.

The C-protein recognition helix (residues 28–40) undergoes a conformational change upon DNA binding (Fig. 5 ▶). The maximum DNA-induced displacement of the main-chain atoms in this region is 2.8 Å. The conformation of the C-terminal region also changes in the protein–DNA complex, but this is most likely to be due to conformational flexibility rather than to any effect of DNA binding. A similar conformational change involving the recognition helix was also observed in C.Esp1396I protein–DNA complexes, but in this case the displacement upon DNA binding was ∼1.4 Å (McGeehan *et al.*, 2012 ▶; Ball *et al.*, 2012 ▶). The interface area between the DNA duplex and the protein dimer is 1424 Å^2^, which is comparable to the values found for C.Esp1396I complexes (1541–1517 Å^2^).

### 4.3. DNA recognition   

**Figure 6:**
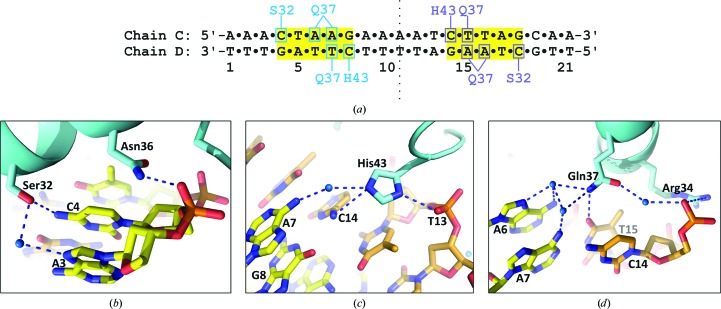
DNA–protein interface. (*a*) Schematic representation of the interactions responsible for DNA recognition at the interface of the protein–DNA complex. (*b*) Detailed view of the main DNA–protein contacts identified in (*a*), including key water-mediated hydrogen bonds.

There are clear contacts to the bases that form the recognition site from the side chains of Ser32, Gln37 and His43 in the DNA–protein complex structure (Fig. 6 ▶). All three amino-acid side chains are also involved in a network of water-mediated hydrogen bonds to additional bases and/or phosphate groups on the DNA, further stabilizing the complex. There are no base-specific contacts with A9/T13 in O_R_ (or the equivalent G9/C13 in O_L_), which is the only site within the 15 bp core sequence that differs between the two binding sites (see Fig. 1 ▶). The same is true for the A2/T20 (C2/G20 in O_L_) and C19/G3 (T19/A3 in O_L_) base pairs that lie outside the recognition site. This is consistent with the DNA-binding affinity at theses two sites being effectively identical. This is in stark contrast to the situation for C.Esp1396I, where the *K*_d_ value for the O_L_ and O_R_ operator sequences differ by many orders of magnitude, consistent with the variation in base sequences at the sites where base-specific contacts are made.

We note that the Ser32, Gln37 and His43 amino-acid residues involved in DNA-sequence recognition are identical in the amino-acid sequence of C.EcoO109I. Furthermore, the nine charged and/or polar amino-acid residues that can be seen contacting the phosphate groups of the DNA backbone in the C.Csp231I DNA–protein complex are also identically located in C.EcoO109I. Thus, there can be little doubt that the latter protein will interact with the DNA-recognition site in the same manner as we see here.

### 4.4. Interactions between adjacent dimers at the O_L_ and O_R_ operator sites   

In common with most other C-protein systems, there are two C-protein binding sites upstream of the C.Csp231I gene. In other systems that have been studied in detail, the left-hand operator (O_L_) distal to the gene is responsible for enhancing transcription of the C-gene by recruiting RNA polymerase; in contrast, the right-hand operator (O_R_) proximal to the gene represses transcription by sterically blocking RNA polymerase. The O_R_ binding site is only occupied at elevated concentrations of C-protein, at which point it switches the gene off to avoid overproduction of the C-protein (and the co-transcribed endonuclease). For C.Csp231I, however, the DNA-binding affinities for the two sites are effectively identical and there is no obvious cooperativity between the two binding sites, and thus we conclude that a different mechanism must be in operation. Moreover, the binding sites are separated by 15 bp (∼60 Å of extended B-form DNA), a distance that is far too great to allow two protein dimers to contact in a side-by-side fashion as seen in other C-protein complexes. If they do contact each other, then the dimers must interact in a ‘back-to-back’ fashion, which implies DNA looping.

Our EMSA experiments show that the length of the DNA spacer (but not its sequence) is critical for forming the tetrameric complex, and thus the two bound dimers most likely interact with each other by looping and folding back of the intervening spacer DNA between bound dimers, rather than adopting an extended conformation. [If there were no interactions between the two dimers bound to the O_L_ and O_R_ sites (*i.e.* if they bound independently), then changing the spacer length should not affect formation of the tetrameric complex.]

As an additional test of whether the tetrameric complex was in a compact or an extended linear structure, we performed sedimentation-velocity analysis by analytical ultracentrifugation (AUC). For comparison, we also performed an equivalent run on the free DNA. The results show that the tetrameric complex has the expected molecular mass of ∼84 kDa and a sedimentation coefficient of 6.14 S indicative of a compact structure (see Supplementary Fig. S3).

**Figure 7:**
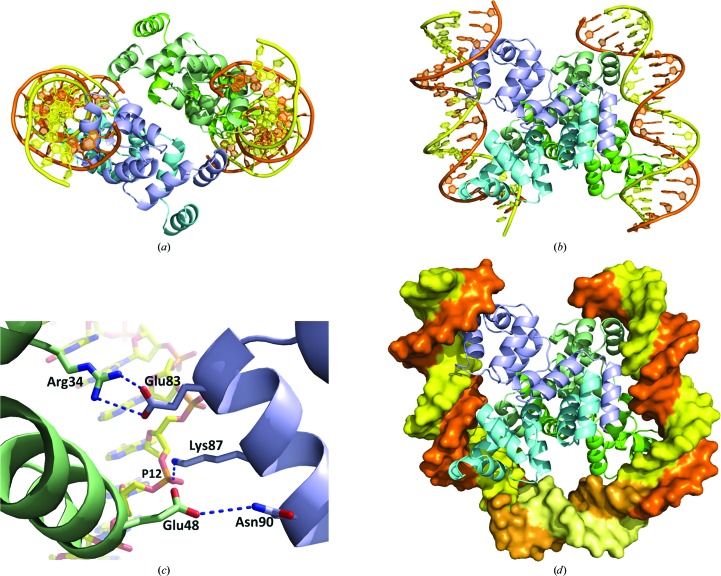
Dimer–dimer interactions and tetrameric model. (*a*) Two dimers are present in the asymmetric unit of the *C*2 crystal and are shown here as ribbon diagrams with their respective DNA operators. (*b*) An orthogonal view of the model in (*a*). (*c*) A detailed view of the interacting residues observed between chain *B* of one dimer and chain *F* of the adjacent dimer. There is also a contact from chain *B* of one dimer to the phosphate group of the DNA bound to the adjacent dimer (Lys87–P12). (*d*) Based on these contacts, a 12 bp spacer was modelled in to form a loop. The DNA is rendered as a space-filling cartoon in the same orientation as in (*b*), with the crystallographic model in dark orange/yellow and the predicted location of the 12 bp spacer in light orange/yellow.

One interesting possibility is that the two dimeric complexes found in the asymmetric unit of the *C*2 crystal form of the complex (Figs. 7 ▶ *a* and 7 ▶ *b*) represent such an interaction but lacking a covalently linked DNA spacer. The two dimeric complexes in the asymmetric unit of the *C*2 crystal (Figs. 7 ▶ *a* and 7 ▶ *b*) are held together by protein–protein interactions between adjacent dimers (Fig. 7 ▶ *c*). Following the notation used in the PDB (entry 4jqd), the dimer at the first site is represented by protein subunits *A* and *B* and the two DNA strands in the complex are labelled *G* and *H*. Likewise, the subunits of the second complex are labelled *E* and *F* and the associated DNA strands *C* and *D*. There are clear contacts between adjacent protein dimers in this tetrameric assembly, including a number of ion-pair interactions, in which Asn90 and Glu83 of subunit *B* in one dimer contact Glu48 and Arg34 (respectively) of subunit *F* in the adjacent protein dimer. In addition, Lys40 of subunit *A* is in proximity to Glu83 of subunit *F*, and these two residues may also interact. There are also potential contacts between a protein subunit of one dimer and the DNA bound to the second dimer; for example, the interaction of Lys87 (subunit *B*) with a phosphate of the DNA (strand *H*). We note that three of these interacting residues (Glu83, Lys87 and Asn90) are located in helix 7 at the C-terminus of the protein. This C-terminal region of the amino-acid sequence is rich in basic amino acids (seven from 13 residues, including three arginines and four lysines). This helix is not found in typical C-proteins, suggesting that it has a unique function that may promote protein–protein and protein–DNA interactions to stabilize the tetrameric complex.

Such interactions between the two complexes could stabilize a looped tetrameric complex. Fig. 7 ▶(*d*) shows a model of such a complex with a 54 bp sequence. It has been constructed by inserting a 12 bp highly curved segment of DNA to link the two 21 bp duplexes of the two complexes in the asymmetric unit. In this model, there would be no sequence-specific interactions from the C-protein to the spacer DNA, consistent with the EMSA results showing that mutating the spacer sequence had no observable effect on DNA binding. It would also explain why the length of the DNA spacer is important, as shorter sequences would be unable to span the gap between adjacent dimeric complexes.

Using the *HYDROPRO* computer program (Ortega *et al.*, 2011 ▶), we can predict the sedimentation coefficient of the tetrameric structure that we propose. The calculated value for the model (6.60 S) is close to the experimental value (6.14 S), adding further support for the looped-back model of the protein–DNA complex. It should be emphasized that the dimer–dimer interactions in our proposed model are stabilized only by the tethering of two dimers when bound to the native 56 bp DNA sequence with an appropriate distance between dimer binding sites, as indicated by our EMSA experiments. For the free unbound protein, we found no evidence of tetramer formation by AUC (McGeehan *et al.*, 2011 ▶).

The proposed U-turn bend induced by the inserted 12 bp spacer is an unusual DNA conformation. However, similar DNA conformations have been observed for structures of *Escherichia coli* IHF (integration host factor) and HU (histone-like protein) proteins (Swinger & Rice, 2004 ▶), the eubacterial integration host Hbb factor from *Borrelia burgdorferi* (Mouw & Rice, 2007 ▶) and the human mitochondrial transcription and packaging factor Tfam (Rubio-Cosials *et al.*, 2011 ▶; Ngo *et al.*, 2011 ▶). The degree of DNA distortion is dramatic in the cases of the Hbb and Tfam protein–DNA complexes, causing an overall DNA bend of ∼180° and a reversal in the direction of the DNA helical axis.

Such a ‘folded-back’ structure for the DNA complex when both left and right operators are bound is very different to the only experimentally determined tetrameric C-protein–DNA complex structure, that of the controller protein C.Esp136I bound to the 35 bp upstream DNA fragment containing both operator sites (McGeehan *et al.*, 2008 ▶), which is essentially an extended linear structure (with some local bending at each dimer binding site). C.Csp231I (together with C.EcoO1019I) represents a unique family of C-proteins, and their amino-acid sequences and DNA-recognition sites are very different from those previously studied. Given the quite different DNA-recognition modes employed by these two C-proteins, as well as the much greater length of the spacer between the dimer binding sites, we expect that their mechanisms of gene activation and/or repression will be very different from other known systems, as exemplified by C.Esp136I.

## Supplementary Material

PDB reference: C.Csp231I–DNA complexes, 4jcx

PDB reference: 4jcy

PDB reference: 4jqd

Supporting Information.. DOI: 10.1107/S139900471402690X/cb5069sup1.pdf

## Figures and Tables

**Table 1:** Crystal, data-collection and refinement parameters Values in parentheses are for the highest resolution shell.

	O_L_1 (hexagonal)	O_L_2 (monoclinic)	O_R_ (hexagonal)
Crystal parameters			
Space group	*P*6_1_	*C*2	*P*6_1_
Unit-cell parameters			
*a* ()	62.2	82.1	62.3
*b* ()	62.2	128.1	62.3
*c* ()	147.8	78.5	158.1
()	90.0	90.0	90.0
()	90.0	100.0	90.0
()	120.0	90.0	120.0
Molecules in asymmetric unit (proteinDNA complexes)	1	2	1
Data collection			
Resolution ()	50.62.3 (2.392.30)	36.02.75 (2.942.75)	54.01.8 (1.901.80)
No. of measured reflections	86190 (10042)	78708 (10482)	134209 (18969)
No. of unique reflections	14402 (1642)	20336 (3378)	32019 (4703)
Completeness (%)	99.9 (100.0)	97.6 (89.0)	99.5 (99.8)
*I*/(*I*)	10.5 (3.2)	9.0 (2.9)	12.8 (2.6)
Multiplicity	6.0 (6.1)	3.9 (3.1)	4.2 (4.0)
*R*_merge_ † (%)	11.1 (55.7)	7.5 (30.4)	5.9 (55.1)
Refinement parameters			
*R*_work_/*R*_free_ ‡ (%)	17.2/19.6	20.5/23.5	13.5/14.8
No. of atoms			
Protein	1541	3085	1494
DNA	855	1710	855
Water	57	51	404
Iodide ions			2
*B* factors (^2^)			
Protein	17.7	19.9	20.3
DNA	16.8	19.4	19.4
Water	34.5	40.4	33.6
Iodide ions			23.9
Average *B* factor	17.8	19.8	22.0
From Wilson plot	29.7	43.3	22.4
R.m.s. deviations			
Bond lengths ()	0.0059	0.0125	0.0041
Bond angles ()	1.058	0.8628	0.9320
Coordinate error§ ()	0.04	0.33	0.02
Ramachandran statistics			
Favoured (%)	96.5	97.7	98.8
Allowed (%)	3.5	2.3	1.2
Outliers (%)	0	0	0

† *R*_merge_ = 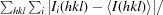 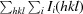, where *I*(*hkl*) is the mean intensity of reflection *hkl* and *I_i_*(*hkl*) is the intensity of an individual measurement of reflection *hkl*.

‡ *R*_work_ = 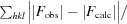 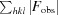, where *F*_obs_ is the observed structure-factor amplitude and *F*_calc_ is the calculated structure-factor amplitude. *R*_free_ is the same as *R*_work_ but for the 5% of structure-factor amplitudes that were set aside during refinement.

§ Estimation based on the *R*_free_ value (estimated by *REFMAC*).
