# Supplementary material for: Structural analysis of DNA binding by C.Csp231I, a member of a novel class of R-M controller proteins regulating gene expression
Source: Acta Crystallogr D Biol Crystallogr. 2015 Jan 23;71(Pt 2):398–407. doi: 10.1107/S139900471402690X (PMC4321490; doi:10.1107/S139900471402690X)
Supplement: Supplementary file 1 [file d-71-00398-sup1.pdf]

BIOLOGICAL CRYSTALLOGRAPHY

Volume 71 (2015)

Supporting information for article:

Structural analysis of DNA binding by C.Csp231I, a novel class of R-M controller proteins regulating gene expression

M. B. Shevtsov, S. D. Streeter, S.-J. Thresh, A. Swiderska, J. E. McGeehan and G. G. Kneale

S1. Location of Iodine Ions

Analysis of the anomalous difference map for the O_R 21-mer complex revealed the presence of two significant peaks, one per protein monomer, with their positions symmetrically arranged around the two-fold rotational symmetry axis within the protein dimer. Both peaks appear at a level of 10σ and are located in well-ordered parts of the structure. The composition of the crystallisation solution (0.2 M sodium iodide) and presence of an anomalous signal at the 0.9795 Å wavelength suggest that these peaks correspond to iodine ions. The ions are bound to the protein surface and surrounded by side chains of residues Leu2, Arg4, Arg5, Asp69 and the main chain of Pro67 (Supplementary Fig. S1). The refinement of iodine ions was done with partial occupancy 0.5 and resulted in temperature factors that were in good correspondence with the temperature factors of surrounding protein atoms, further supporting assignment of the peaks to iodines.

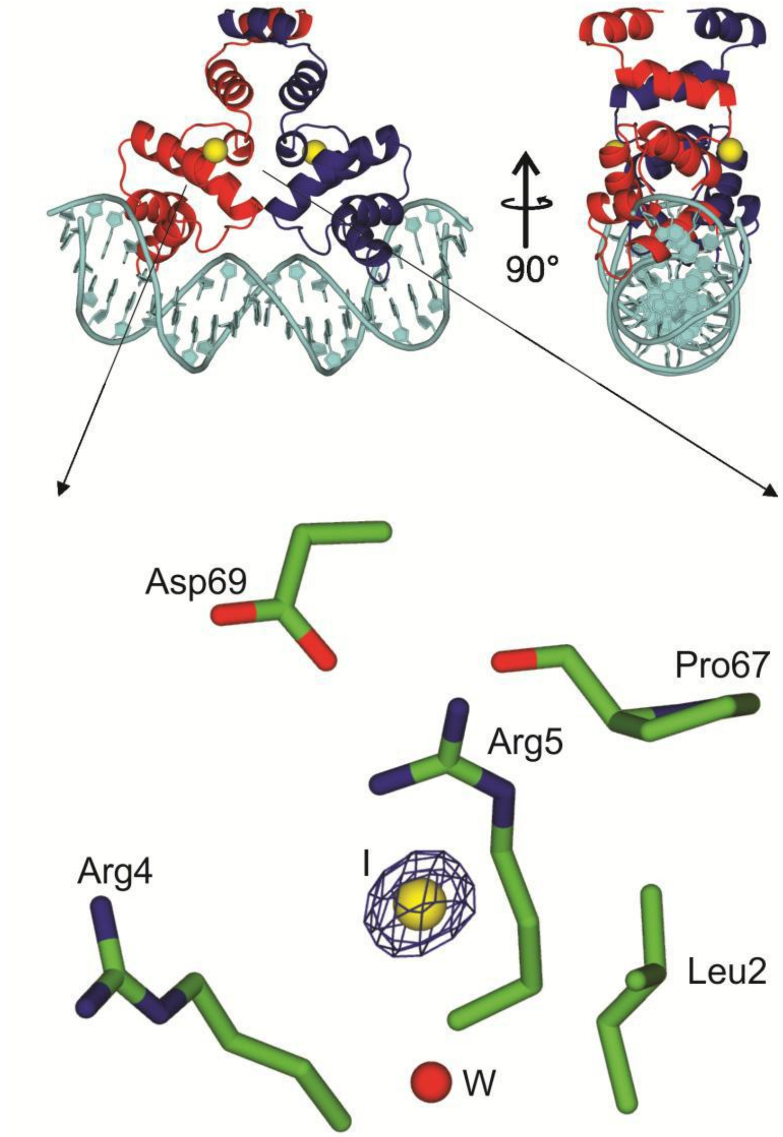

Figure S1 Iodine binding sites of the 21-mer O_R complex: (A) The protein dimer (red and blue) bound to the DNA duplex (cyan) is shown in two orientations. Two iodine ions are shown by yellow spheres. (B) The anomalous difference map is contoured at level 5σ and shown in blue. Surrounding residues within a 4.8 Å radius are shown. The water molecule in proximity of the binding site is shown by red sphere.

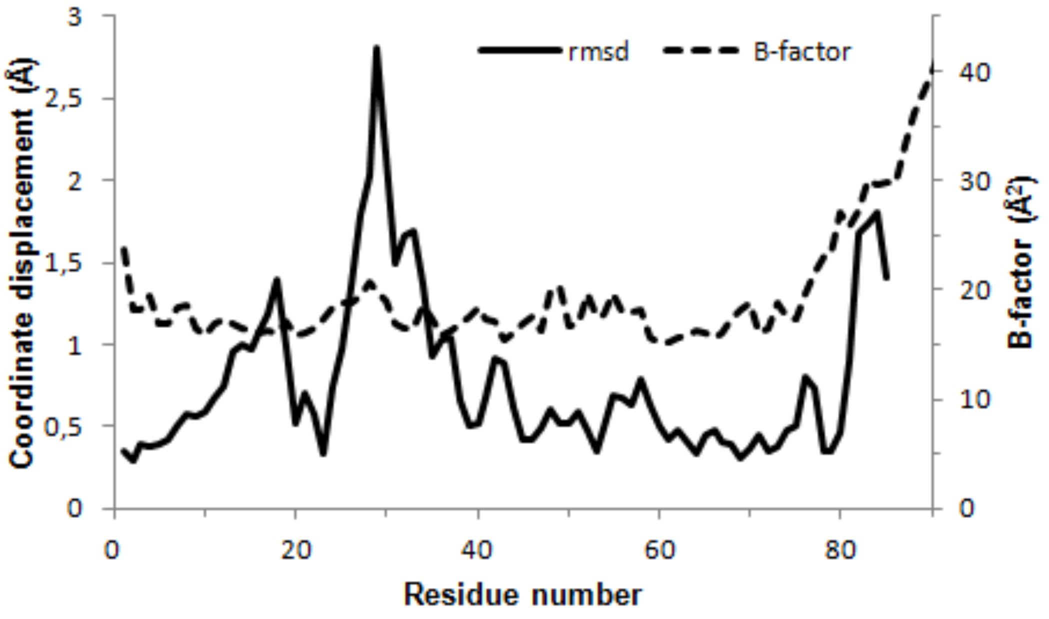

Supplementary Figure S2

(a) RMS deviation profiles showing differences between DNA-bound and free protein structures along the amino acid sequence of the protein (full line).

(b) Average B- factor per residue showing conformational flexibility at the C-terminal region of the protein (dotted line).

(a) 56 bp DNA S= 3.66 S; M=34,320 f/fo=2.15

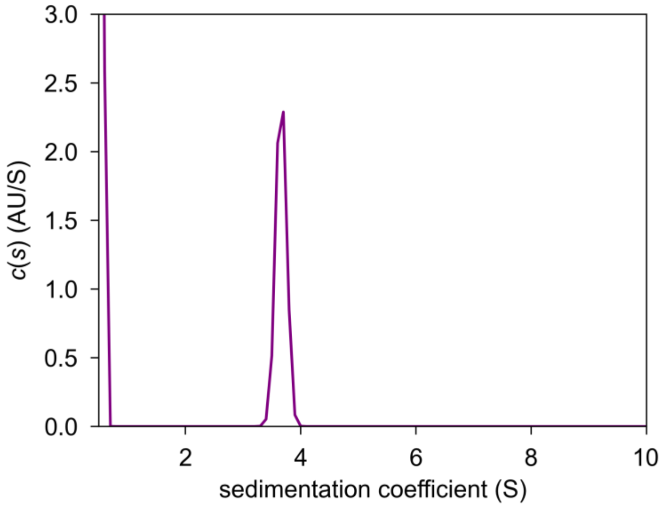

(b) 56 bp DNA / protein complex: 2 C-protein dimers/duplex

S=6.14 S; M=82,784 f/fo =1.53

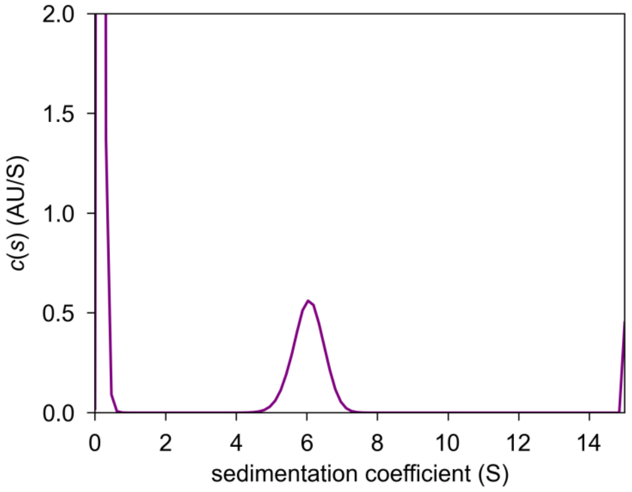

Figure S3 Sedimentation Velocity Analysis of DNA and the C.Csp231I-DNA complex. Samples were equilibrated at 20 °C for 30 min and then accelerated to 20,000 rpm. Radial scans were performed at 10 min intervals at 260 nm. The DNA concentration was 0.76 µM and for the tetrameric complex, the protein was at a 4:1 molar ratio (subunits per DNA duplex). The partial specific volume for C.Csp231I was calculated from the amino acid composition using SEDNTERP (Hayes *et al.*, 2006) at 0.7448 ml g^{-1}, with a buffer density of 1.00283 g ml^{-1} and a viscosity of 0.010137 P. Analysis of the scans was performed using the program SEDFIT [Schuck, 2000] to produce a distribution plot (c(S)) of the sedimentation coefficient profile.
